# Minimally Invasive Hemostatic Materials: Tackling a Dilemma of Fluidity and Adhesion by Photopolymerization *in situ*

**DOI:** 10.1038/s41598-017-15368-8

**Published:** 2017-11-10

**Authors:** Yun Zhang, Dandan Song, Hong Huang, Zhiling Liang, Houhe Liu, Yugang Huang, Cheng Zhong, Guodong Ye

**Affiliations:** 1 The Fifth Affiliated Hospital of Guangzhou Medical University, Guangzhou 510275, and Key Laboratory of Molecular Clinical Pharmacology, School of Pharmaceutical Sciences, Guangzhou Medical University, Guangzhou, 511436 People’s Republic of China; 2 College of Chemistry and Molecular Sciences, Wuhan University, Wuhan, 430072 People’s Republic of China

**Keywords:** Photochemistry, Quantum chemistry

## Abstract

Hemostasis *in vivo* is a key to success in minimally invasive surgery (MIS). However, solid hemostatic materials cannot pass through the sheath tube of the MIS apparatus, while liquid ones are restricted by their low adhesion, which leads to them peeling off of tissue. To tackle the dilemma of fluidity and adhesion, a formulation containing a multifunctional sucrose allyl ether (SAE) monomer and an alpha-hydroxyketone liquid photoinitiator (HMPP) was applied as a lead hemostatic material for MIS. Real-time infrared results showed that SAE initiated by HMPP can rapidly polymerize into a transparent crosslinking membrane. Quantum chemistry showed that this occurs via a free radical addition reaction mechanism. Thermodynamic properties, such as reaction driving force and enthalpy change, were similar to those for a corresponding small molecular analogue, allyl methyl ether (AME), but the addition rate was lower than that for AME. The CC50 values of SAE and HMPP were also obtained by cell experiments. A hemostasis experiment *in vivo* was performed by comparing the formulation with chitosan and a traditional Chinese medicine (Yunnan Baiyao powder). The result showed that the formulation had a competitive advantage for use in MIS.

## Introduction

Minimally invasive surgery (MIS) is a method of surgical treatment with minimum trauma using a laparoscope, a thoracoscope, an intranasal endoscope and other medical equipment^1,2^. A surgeon often encounters internal haemorrhage in MIS. Therefore, hemostasis is key to surgical success. If the ultrasonically activated scalpel or electrotome cannot seal blood vessels, it will cause haematoma and require a second surgery, or a life-threatening complication will occur^3^.

A series of hemostatic materials have been developed, e.g., thrombin and fibrinogen in polypeptide protein. Other macromolecular materials include natural materials (e.g., oxidized cellulose and chitosan) and polymeric materials (e.g., polyvinyl alcohol and polylactic acid). These materials are very applicable to traditional surgery considering their acceptable biodegradability and good biocompatibility^4,5^. However, they generally form a membrane *in vitro*^6^, highlighting the lack of liquidity when used in MIS; in a surgical procedure, a surgeon makes several small (<1 inch) incisions and inserts a trocar into the patient’s body. A low viscosity material will be necessary for microchannel transport for application to the affected part. Solid materials, such as polylactic acid (PLA), cannot meet this requirement, while liquid materials, such as polyvinyl alcohol (PVA), do not stick to tissue. Other monomers, such as cyanoacrylate, often blocked the trocars/micro-catheters, and lauromacrogol can easily result in a sclerotic tissue. Thus, we proposed to tackle the dilemma of fluidity and adhesion using photopolymerization *in situ*^7^.

The formulation is typically composed of a monomer and a photoinitiator (PI). Here, sucrose allyl ether (SAE) is selected as a lead monomer because of its biosafety and fluidity. Although a monosaccharide type of glycoside, such as aminoglycoside, is common in many plants^8–10^, it has been reported that these glycosides have a certain toxicity. Acrylate-based monomers, such as polyhydroxyethyl methylacrylate, appear to be difficult to maintain as coagulation factors in a gel due to their tightly formed membranes, although hydroxyethyl methylacrylate can undergo rapid polymerization. Another disadvantage is that propionic-like acids are a decomposition product of these membranes, which will lead to or exacerbate inflammation similar to a polylactic acid. SAE has good hydrophilicity in terms of its medium hydrophilic-lipophilic balance value and easily adheres to soft tissue. In addition, SAE is structurally based on sucrose and can thus be considered a safe biomaterial. When SAE is polymerized into a cross-linking membrane, the residue allyl ether can be converted into polyallyl alcohol if the ether group is hydrolysed. Polyallyl alcohol can be considered a polyvinyl alcohol analogue. The published experience in patients and the literature suggest that polyvinyl alcohol as a pharmaceutic adjuvant or an embolic material is safe, without obvious long-term side effects. Based on an overall analysis of the reactivity, biosafety and fluidity, SAE is selected as an initial hemostat used in MIS. In addition, a photoinitiator and a monomer must be quickly mixed on-site in an emergency situation. A pre-mixed formulation seems to be inefficient because of dark polymerization. Hence we chose a liquid PI, 2-hydroxy-2,2-dimethylacetophenone (HMPP), to initiate polymerization for practical reasons.

Therefore, as we intend to use SAE as a lead hemostatic material, we optimized the polymerization conditions and explored the polymerization mechanism using transition state theory. A series of biological safety-related performance tests, i.e., cytotoxicity and the hemostatic effect, were also evaluated. Sucrose oleate (O-170) as a monomer and ethyl-2,4,6-Trimethylbenzoylphenylphosphinate (TPO-L) as a photoinitiator were selected for comparison.

## Results and Discussion

### Synthesis and characterization of SAE

**Figure 1:**
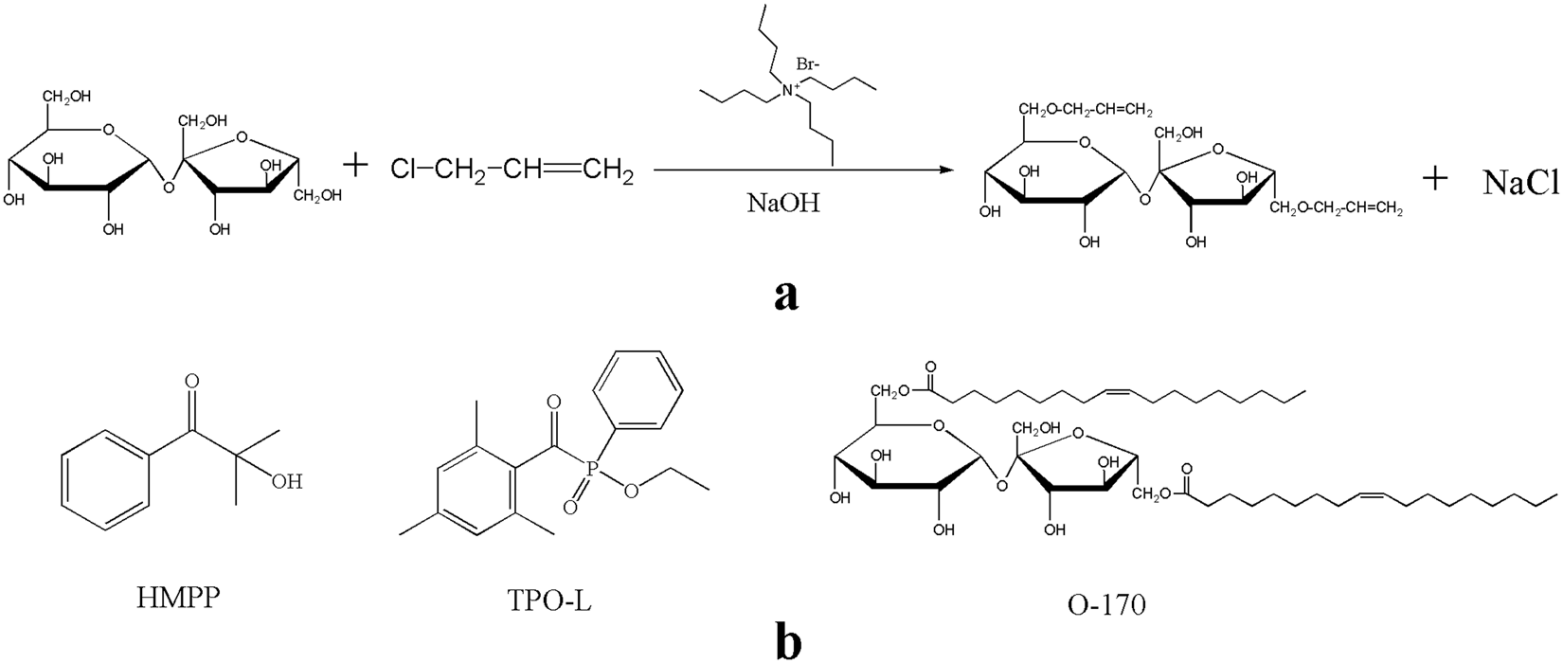
Structure of compound and reaction route. Part (**a**) synthetic path of SAE; part (**b**) structure of photoinitiators and monomer.

The SAE synthetic route is shown in Fig. 1. Sucrose (500 g), sodium hydroxide (750 g), allyl chloride (1032 g), tetrabutylammonium bromide (24 g) and distilled water (600 g) were placed in a reaction kettle and stirred at 75 °C for 15 hours. After the reaction, sodium chloride was removed by centrifugation, and equal volumes of water and ethyl acetate were added to the filtrate; 20% aqueous hydrochloric acid was added to neutralize the solution to approximately 7. To remove the aqueous phase and the upper oil phase, the solution was washed twice. The solution was distilled at 100 °C for 2 hours under reduced pressure to depress the molecular weight and remove ethyl acetate. Then, 2% diatomaceous earth was added and stirred at 100 °C for 1.5 hours. In total, 343 g purified SAE was obtained, in a yield of 33.2% (calculated from the amount of allyl chloride feed).

We found that the multisubstituted sucrose monomer is a liquid, while the monosubstituted monomer is a solid. Thus, the multisubstituted product is suitable for potential application in MIS. Unfortunately, the multisubstituted product is mixed with bisubstituted and trisubstituted sucrose monomer. The chemical environments of H atoms in the sucrose ring are similar, so the numbers of double bond substitutions are difficult to deduce from the integration of H-NMR peaks. Thus, the number of double bonds was detected as approximately 2–3 per sucrose molecule according to iodometry tests. The characterization is shown below: FT-IR (KBr pellet, cm^−1^): 1003 (ν C-O-C adjacent to the five-membered ring and six-membered rings), 1080 (ν C-O-C adjacent to the C=C group), 1644 (ν C=C), 3210 (ν OH); ^1^H NMR (500 MHz, CDCl_3_, δ ppm): 4.02 (d, 4 H, CH_2_ on the ethylene group), 5.91 (m, 2 H, CH on the ethylene group), 5.26 (d, 4 H, aliphatic CH_2_ adjacent to the C=C group); ^13^C NMR (125 MHz, C DCl_3_, δ ppm): 129.06 (CH_2_ on the ethylene group), 129.06 (CH on the ethylene group), 77.04 (aliphatic CH_2_ adjacent to the O-C-C=C group), 117.40 (five-membered ring CH near the C=C group).

**Figure 2:**
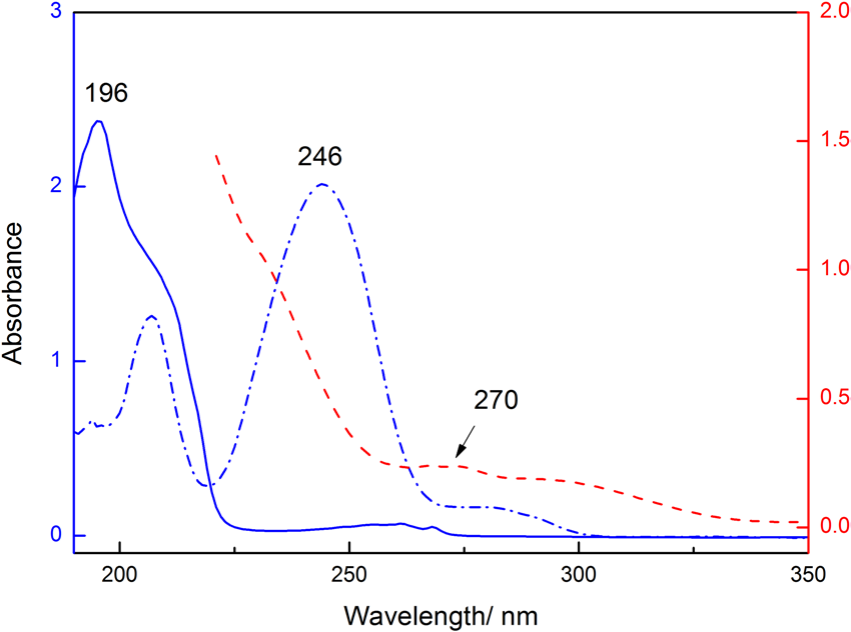
UV-vis spectra of the sucrose monomer and liquid photoinitiators in CH_2_Cl_2_, SAE (5.4 × 10^−3^ M, ), HMPP (1.0 × 10^−4^ M, ), and TPO-L (1.0 × 10^−4^ M, ).

Figure 2 shows the UV-*vis* absorbance characteristics of the SAE monomer, the photoinitiator HMPP and the photoinitiator TPO-L. Their absorption maxima are, respectively, at 196 nm (*ε* = 4.39 × 10^2^), 246 nm (*ε* = 1.9 × 10^4^) and 270 nm (*ε* = 2.36 × 10^3^). The λ_max_ of the sucrose monomer and photoinitiators do not overlap, which will thus not cause competitive absorption and benefit the photoinitiation reaction^11^.

**Figure 3:**
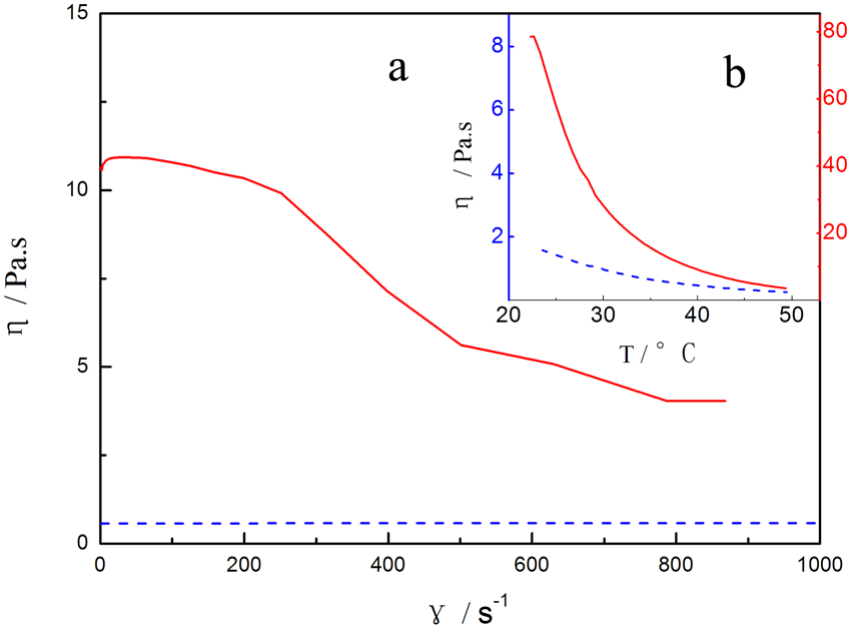
Viscosity (*η*) of SAE () and O-170 (). Part (**a**) viscosity in shear rate (γ) sweep at 37 °C. Part (**b**) viscosity-temperature curve at a shear pressure of 1 Pa.

Since MIS mostly uses an injection tool, it is necessary to study SAE’s viscosity. The π bond of O-170, which is substituted at the sucrose ring by oleoyl groups, is in the middle of the oleoyl chain, while that of SAE is at the end of the allyl chain. The temperature of the liquid used in MIS is between 20 and 45 °C. Figure 3b shows the viscosity changes from 20–50 °C. Both the viscosity of SAE and O-170 decrease with increasing temperature, but that of SAE decreases much faster than that of O-170. The two viscosity-temperature curves approach nearly the same lowest value at approximately 37 °C, which is the temperature of the human body. As seen in part a, the viscosity of SAE decreases with increasing shear rate^12^. However, the viscosity of O-170 was not influenced by the shear rate. A long branch of O-170 can encapsulate the hydroxyl groups in the sucrose molecule; this can explain why the viscosity of O-170 is generally lower than that of SAE. Since a series of micro-injection devices is used in MIS, the result of the shear rate shows that mild external pressure and appropriate temperature control can allow SAE to flow and be sprayed to the bleeding site.

### Kinetic study

Real-time Fourier transform infrared (RT-IR) was used to obtain a kinetic profile of photopolymerization. In MIS, i.e., enteroscopy, CO_2_ is often used. Thus, radical polymerization can be used in MIS without obvious oxygen inhibition. The film was found to be relatively transparent, which is very conducive for a doctor to observe the therapeutic effect during surgery. This is superior to other opaque materials such as chitosan.

**Figure 4:**
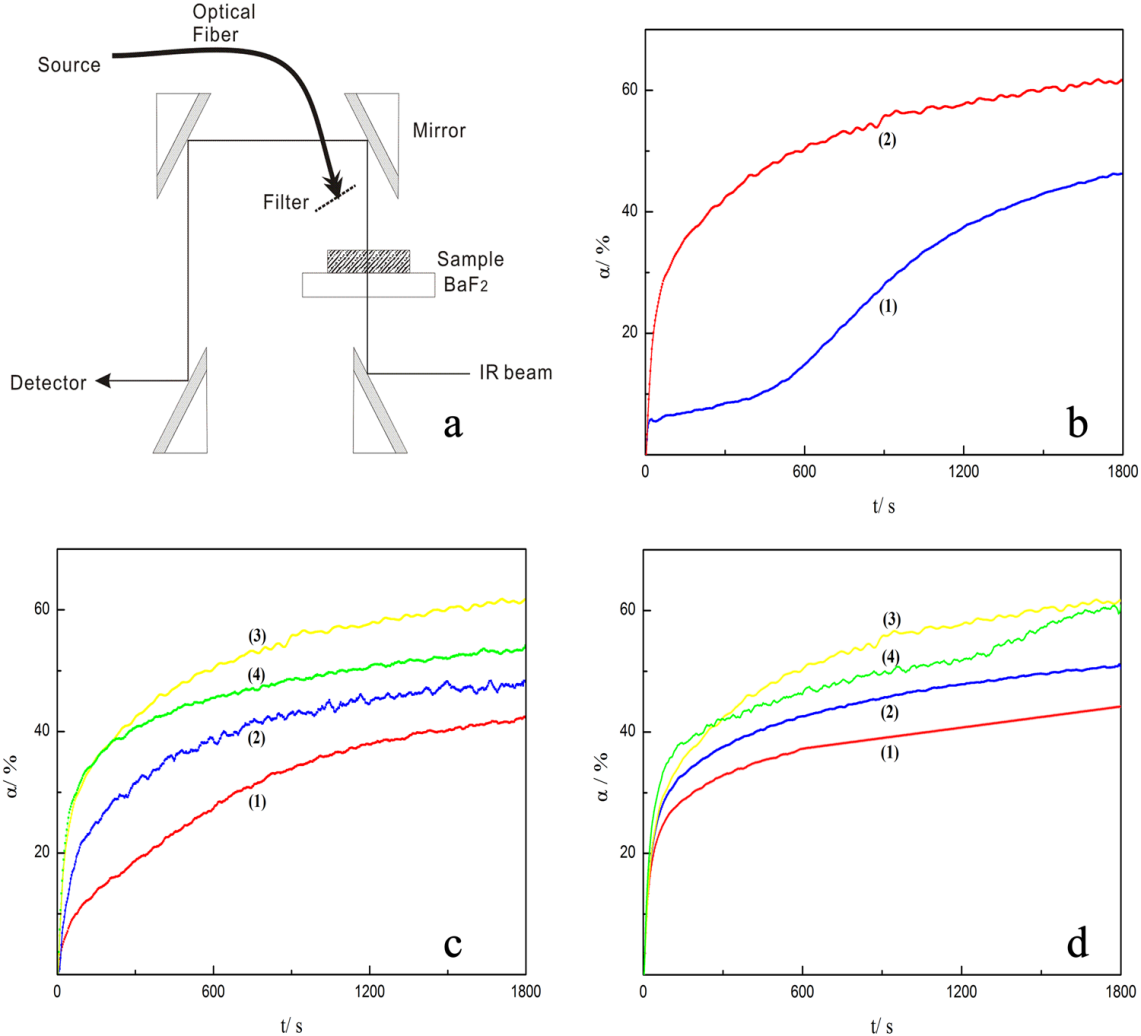
Kinetic investigation of hemostatic material. Part (**a**) diagram of the RT-IR apparatus; part (**b**) plots of *α*-t for SAE initiated by (1) 6.00 wt % TPO-L, (2) 5.98 wt % HMPP, 30 °C, light intensity 38 mW.cm^−2^; part (**c**) plots of *α-t* for SAE with HMPP concentrations [*PI*] of (1) 2.01, (2) 4.12, (3) 5.98 and (4) 8.22 wt %, light intensity 48 mW.cm^−2^, 30 °C; part (**d**) plots of *α*-t for SAE with varying light intensity. HMPP concentration [*PI*] 5.98 wt %, 30 °C, light intensity: (1) 28 mW.cm^−2^, (2) 38 mW.cm^−2^, (3) 48 mW.cm^−2^ and (4) 58 mW.cm^−2^.

In the RT-IR, to avoid gravity effects on membrane formation, total infrared reflection equipment was used to orient the sample horizontally for irradiation, as shown in Fig. 4a. To evaluate the initiation efficiency, another commercial liquid photoinitiator with a low molecular weight, TPO-L, was chosen to initiate photopolymerization for comparison, as shown in Fig. 4b. It is well established that the photodecomposition of HMPP and TPO-L follows a Norrish type I mechanism. The final conversion of HMPP is much higher than that of TPO-L. The polymerization rate of TPO-L is slow at the beginning of reaction, leading to a longer induction period for TPO-L than for HMPP. This may be due to the higher excitation energies of TPO-L. This prevents TPO-L from cleaving to/from phosphorus- and carbon-containing radicals quickly and then adding onto a C=C group. Thus, HMPP was selected as the preferred photoinitiator. Its biosecurity will be discussed in the cytotoxicity test section. To evaluate the influence of HMPP concentration on polymerization, four different concentrations were chosen for the comparison, as shown in part c. The final conversion will initially increase with an increase in HMPP concentration to a maximum value but then decrease with a further increase in HMPP. The decrease in the last stage may be caused by excess primary radicals from the cleavage of HMPP. Thus, the optimal concentration of HMPP for the photopolymerization of SAE is approximately 5.98 wt %. Part d also shows that increasing light intensity, to a certain degree, can increase the polymerization rate and final conversion, but once the light is too intense, there is a decreasing tendency for the final conversion. This is also true for a large amount of primary radical presenting in a short time. The light intensity 48 mW.cm^−2^ is most suitable for the light curing; at this intensity, SAE can be completely cured and will not fracture due to high temperature.

### Chemical calculation

To better understand the polymerization mechanism, density functional theory (DFT) was used to obtain thermodynamic properties such as enthalpy change (∆*H*), driving force (∆*G*), and activation energy (*E*_a_). The reaction rate *k*(*T*) was calculated using variational transition state theory as expressed as Equation (1) ^13^:

1 $${k}^{vTST}(T)=\frac{\sigma }{h\beta }\cdot \exp [-\beta \cdot \Delta G(T,\,{s}_{\ast }^{vTST})]$$

where σ is the symmetry number, β = k_b_/T, and ∆G (T, ${s}_{\ast }^{vTST}$) coincides with maximal free energy. The calculations are tabulated in Table 1. For comparison, allyl methyl ether (AME) was reacted with a benzoyl radical and calculated with the same method. As seen in the table, the *E*_a_, and ∆*G* of benzoyl radical added to SAE are nearly the same as those for AME, which means that the space resistance induced by a sucrose ring can be ignored. Both ∆*H* values are negative, indicating that both radical addition reactions are exothermic. However, the ∆*H* of SAE is less than that of AME, indicating that the entropy changes of the two reactions are different and that if SAE is applied to the human body, it will release less heat. Although the thermodynamic properties of SAE reacting with the benzoyl radical are similar to those of AME, the reaction rate for SAE is approximately 25% that of AME. We believe that polymerization will still proceed quickly to yield a hemostatic effect, as confirmed in the following animal experiment. The mechanism of the free radical addition has been thoroughly investigated by our group elsewhere^14^.

**Table 1:** Thermodynamic properties and kinetic data of the free radical addition.

	*E*_a_ (kJ/mol)	∆*H* (kJ/mol)	∆*G* (kJ/mol)	*k*^*v*TST^ (cm^3^/molec/s)
benzoyl-SAE	35.11	−52.12	−5.15	9.7820 × 10^−22^
benzoyl-AME	35.07	−54.74	−5.19	4.5720 × 10^−21^

### Cytotoxicity test

**Figure 5:**
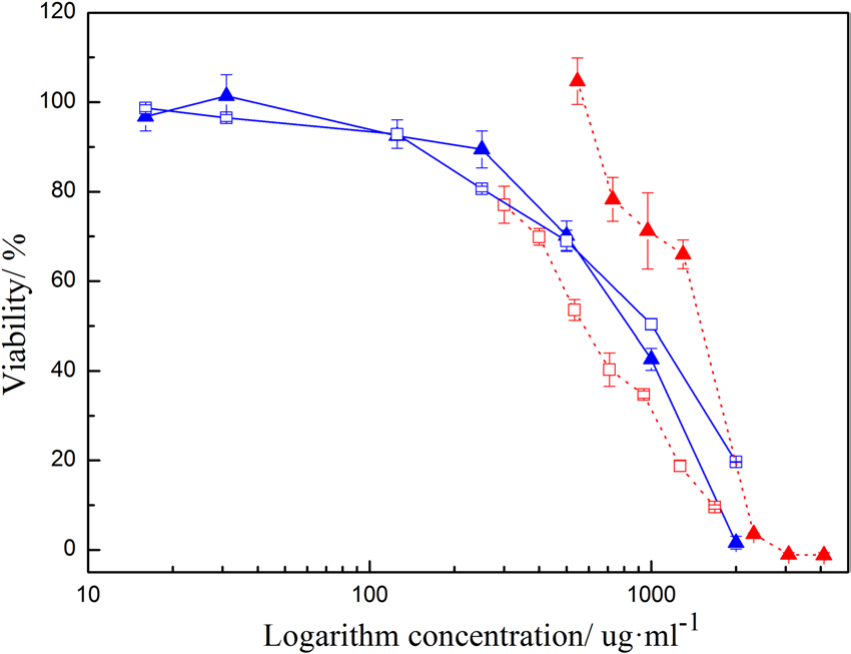
Cytotoxicity of SAE (◽) and HMPP (▴) using the CCK-8 assay with L929 cells (), and BRL 3 A rat liver cells ().

The SAE used in the cytotoxicity test was purified as stated previously. According to ISO 10993-5 and based on our experiment in rat liver, we chose L929 cells and BRL 3 A cells to evaluate the cytotoxicity of compounds in the formula^15,16^. The viability results of a 24 h incubation with different concentrations of SAE or HMPP are shown in Fig. 5. In cell viability studies with the CCK-8 assay, lower CC50 indicates severe cytotoxicity.

The result shows that the CC50 values for L929 cells of SAE and HMPP were 855 µg/ml and 696 µg/ml, respectively. The CC50 values for BRL 3 A cells of SAE and HMPP were 594 µg/ml and 1243 µg/ml respectively. Compared to other photoinitiators, such as alpha-aminoketones (Irgacure 907), whose CC50 was 149 µg/ml on human monocytes, HMPP shows lower cytotoxicity^17^. Eick *et al*. reported that the photoinitiator Sarcat^TM^ CD 1012 had a CC50 of 10 µg/ml, which is very cytotoxic^18^. In another study, the photoinitiating agent 0.03% benzoin alkyl ether (Irgacure 651) was found to completely kill all human foetal osteoblasts and bovine chondrocytes^19^. Although some alpha-hydroxyketones (e.g., Irgacure 2959) have highly satisfactory biosecurity, they should be rejected for use in MIS because of their solid appearance, as mentioned above.

**Figure 6:**
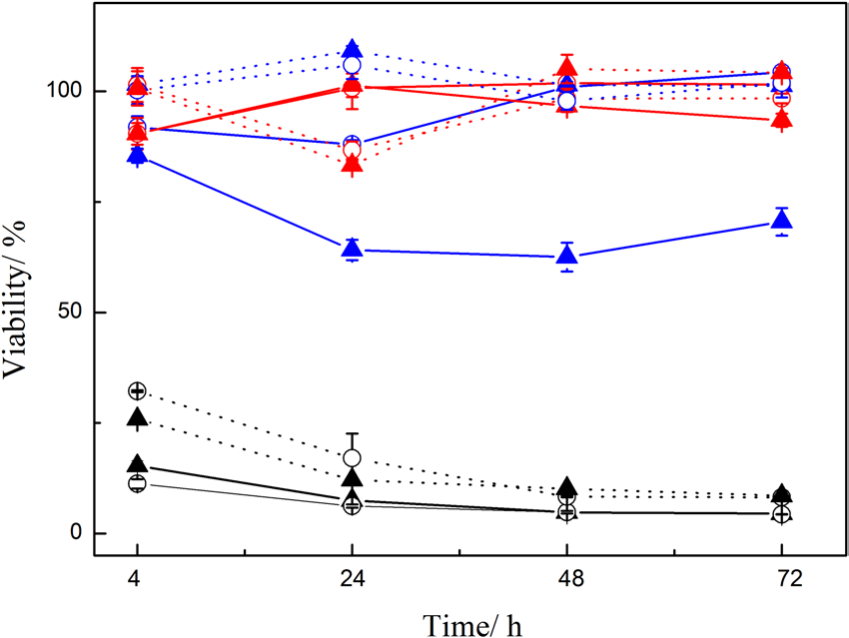
Cytotoxicity of extracts of material. Material was cured with SAE + HMPP (6%) using the CCK-8 assay with L929 cells () and BRL 3 A rat liver cells (); material extracted for 24 h is represented as ⚬ and 72 h as ▴. The samples are in blue, the negative control groups are in red, and the positive control groups are in black.

Figure 6 shows the result of biological assay of the membrane. The extracts at 24 h had no reduction in cell growth, indicating qualitative cytotoxicity of the 24 h extracts for the two kinds of cells of grade 0 level. However, for extracts of 72 h, there was a little reduction in cell growth for BRL 3 A, which means that cytotoxicity was at grade 1 level, and there was approximately 40% cell growth inhibition for L929 cells, with cytotoxicity at grade 2.

### Adhesion ratio

The binding strength created between a film as a hemostat and a meat product as a substrate is greater than the film strength. This means that the peeling strength (adhesive force) is greater than the fracture strength (crosslinking force). This phenomenon is frequently seen with UV curing, and it is difficult to obtain the peeling strength based on the exfoliation properties at the tissue level. Considering the importance of adhesion for a hemostat, we detected the adhesive force with cell adhesion experiments. The result is shown in Table 2: the adhesive force is enormous for both samples at the cell level, confirming the peeling test results indirectly.

**Table 2:** Result of cell adhesion experiments.

Cell	Adhesion rate			Mean
BRL3A	79.97%	74.30%	73.15%	75.80%
HEPG2	87.85%	82.54%	81.40%	83.93%

BRL3A: rat liver cells, HEPG2: human hepatoma cell line.

### Hemostasis experiment *in vivo*

**Figure 7:**
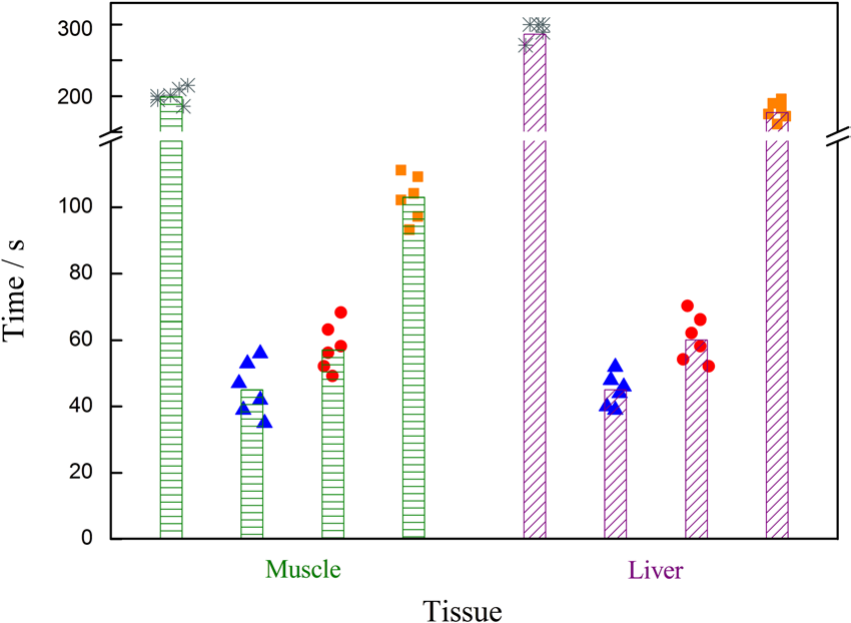
Complete hemostatic time of three hemostatic materials.  Is the blank control,  is SAE + HMPP,  is chitosan powder, and  is YBP powder. The bar chart shows the average time.

The feasibility of SAE for our hemostatic material was confirmed in the above theoretical calculation and experiment. To further investigate its practical applications as a hemostatic material *in vivo*, the formulation was used to cover bleeding wounds and was compared to chitosan and Yunnan Baiyao powder (YBP). Chitosan is a very effective powder for lethal bleeding that has been widely used by the US military^20^. YBP is a well-known Chinese herbal medicine that has been used as a hemostatic drug for more than a century^21^. Compared to SAE, the common advantage of chitosan and YBP is that they can be applied for hemostasis without UV irradiation. The results from RT-IR show that the reaction takes a long time to reach final conversion, but 80% of final conversion occurs after less than 300 seconds. The membrane at the final conversion has a hard, tight structure that may not be suitable for storage of red blood cell/coagulation factors. Thus, it is not necessary for us to convert SAE to a tight membrane via long-time UV irradiation. A coating with a loose structure can more easily stop bleeding via good stagnation. Thus, we take the maximum exposure time within 50 seconds as the experimental irradiation time. Twelve rats received no treatment on their bleeding wounds as a blank control. Figure 7 shows the hemostatic time for various hemostatic materials. The pre-experimental data show that the hemostatic time of the three hemostatic materials is less than 120 s, and thus, it is not necessary to record long hemostatic times in the experiment. Any wound bleeding after 5 mins was recorded as a hemostatic time of 5 mins.

From the above figure, the hemostatic time relationship of the three hemostatic materials is apparent. The result shows that the average hemostasis time of SAE + HMPP is shortest and that of YBP is the longest. The hemostatic times of SAE + HMPP and chitosan are basically the same for different application sites. However, to obtain the same hemostatic effect, YBP applied on the liver should take more time than on muscle. Due to less blood flow, the muscle group without any treatment can self-coagulate given enough time. For the liver, the blank control group still bled for over 5 mins because of the large blood flow.

**Figure 8:**
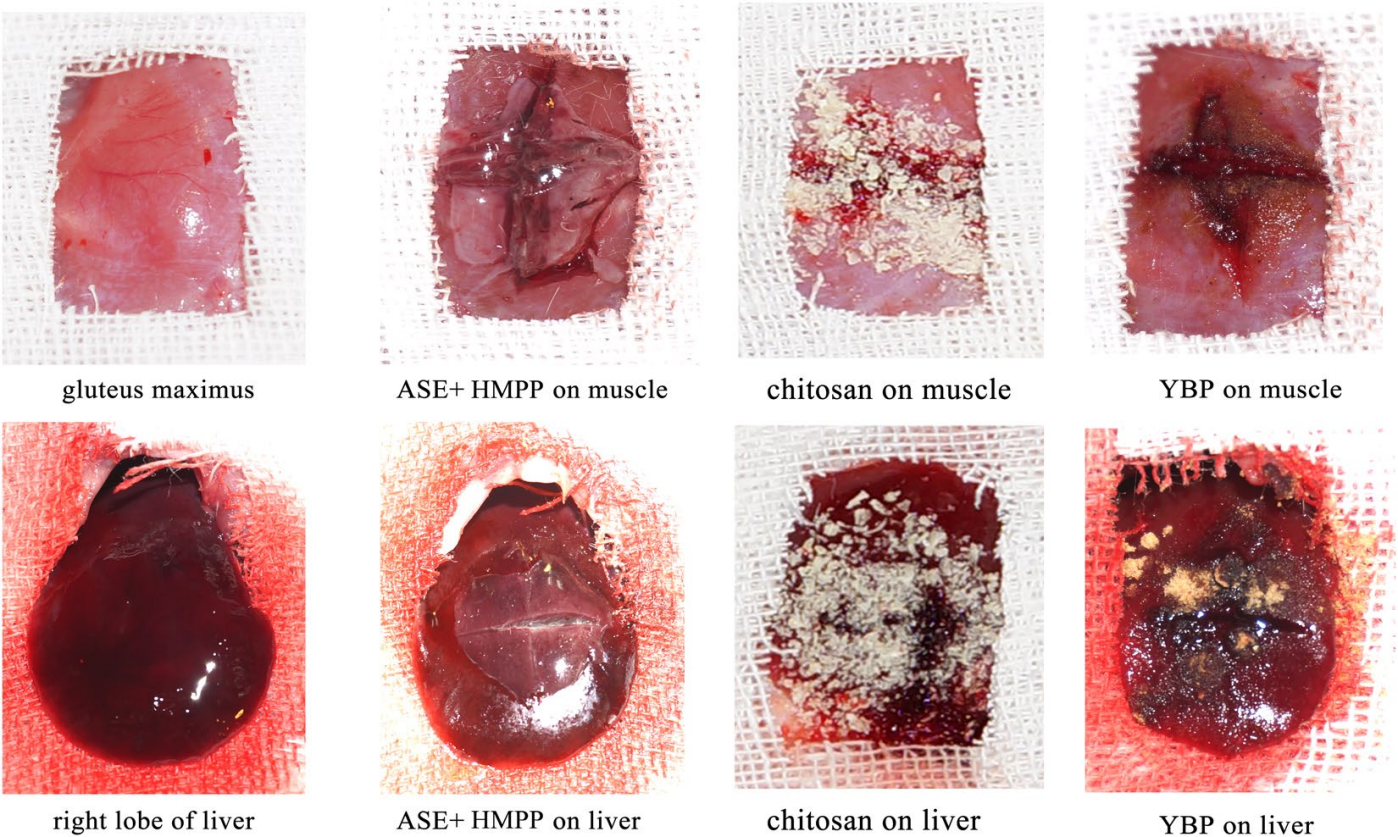
Experimental observation of hemostatic materials.

After cutting the muscle, a small amount of blood oozed out. After coating the muscle with a thin layer of SAE-HMPP complex at the muscle incision and irradiation of UV light, liquid SAE gradually converted into a solid coating. A transparent membrane can help a surgeon to judge the effect of hemostasis. The membrane forms a closed space with the tissue, and hemostasis is observed. The results before and after bleeding can be seen in Fig. 8. The chitosan and YBP powder gradually dissolved after application to the incision; they can be absorbed, resulting in hemostasis. Bleeding was gradually alleviated until it fully stopped. A robust plug was formed when red blood cells reacted with chitosan powder to achieve hemostasis^22^. The hemostatic mechanism of YBP is that the absorbed powder promotes blood coagulation and accelerates wound healing^23^. To better reflect differences in data, hemostatic times were statistically analysed by a randomized block design, with P < 0.01 indicating a very significant difference, P < 0.05 a statistically significant difference and P > 0.05 no significant difference.

Statistical analysis of hemostasis time showed that the standard deviation of the blank control and the three hemostatic materials was less than 12%. The hemostatic time data distribution was concentrated, and the degree of dispersion was low. For muscle, the blank control group showed that rats can stop bleeding via their own blood clotting mechanism due to the low blood flow. However, the liver site cannot stop bleeding via self-coagulation due to the large blood flow. For the SAE + HMPP and chitosan powder groups, in both the muscle group and liver group, their P values were < 0.05 for “Inter-treatment” and > 0.05 for “Interblock”. Thus, the hemostatic effect of the two hemostatic materials is different. The hemostasis time for the SAE + HMPP group was slightly shorter than that for the chitosan powder group. In the same treatment group, there was no difference between the different SD rats. For the SAE + HMPP group and YBP group, both the muscle group and the liver group P values were < 0.01 for “Inter-treatment” and > 0.05 for “Interblock”. Thus, the hemostatic effect of the SAE + HMPP group and YBP group differed very significantly. In the same treatment group, there was no difference between the SD rat groups, indicating that the hemostatic time for SAE was shorter than that for chitosan powder and much faster than that for YBP. The SAE + HMPP treatment took only approximately 45 seconds to stop the bleeding, which would be a great advantage in MIS.

Short-term ultraviolet irradiation of platelets has not been shown to cause obvious clinically significant change in platelet function. Irradiation of red blood cells may reduce cell activity, possibly due to haemoglobin denaturation under the irradiation. According to the literature^24^, ultraviolet-ray irradiation of fresh plasma does not alter its clotting mechanism for the blood clotting factors (prothrombin, factor V and factor VII). Therefore, the possible mechanism is membrane aggregation of red blood cells or platelets. We will continue to investigate this phenomenon in the future for a greater understanding of the mechanism of action of the material.

## Conclusions

The RT-IR experiment shows that a formulation with SAE and HMPP can readily undergo polymerization under irradiation conditions to form a transparent membrane. Optimal concentration and light intensity can increase the rate of polymerization and obtain a satisfactory final conversion. The result of quantum chemistry shows that the *E*_a_ and ∆*G* of the radical addition reaction was the same as those of its corresponding small molecular analogue AME, indicating that this process proceeds by a radical addition reaction mechanism. The ∆*H* of SAE is lower than that of AME, indicating that the reaction releases less heat than that with AME. Cytotoxicity tests verified that the formulation with SAE and HMPP combined had good biosafety according to the CC50 value for BRL 3 A and L929 cells. In the *in vivo* hemostasis experiment, the hemostatic time for SAE-HMPP was much faster than those for chitosan and YBP powder. There was no direct relationship between hemostatic time and blood flow. This suggests the feasibility of using SAE as a minimally invasive material for hemostasis *in vivo*.

## Experimental Section

### Compounds

SAE was synthesized according to the literature^25^. Sucrose, sodium hydroxide, allyl chloride and tetrabutylammonium bromide were of analytical grade and obtained from the Aladdin Industrial Corporation. The concentration of sodium hydroxide was adjusted to 30%–50% before use. The degree of unsaturation of SAE was obtained by iodometry (Wijs method)^26^. HMPP and TPO-L were purchased from Ciba Specialty Chemicals Co., Ltd. and purified by distillation. Pentobarbital sodium as an anaesthetic drug was purchased from Xiya Reagent. Chitosan powder was obtained from MedTrade Products, Ltd. Yunnan Baiyao powder was obtained from Yunnan Baiyao Group Co., Ltd.

### Instruments

UV-vis spectra were obtained with an 8453 UV spectrophotometer (Agilent Co.). Viscosity data were obtained using a Kinexus Pro rotary rheometer (Malvern). NMR data were obtained using a Bruker AVANCE III 500. An LC8 point light source L9588 (Hamamatsu) was used for photopolymerization of SAE, and the light intensity was measured in a UV radiometer sensitive in the wavelength range of 320–400 nm; an FTIR-7600 type Fourier transform infrared spectrometer (Thermo Fisher) was used for the RT-IR experiment. The transmission signal in the infrared was synchronously collected using an automated trigger when the sample was exposed to a spot light source. The light source had a broad UV spectra emission. The reaction was monitored by following the change in the stretching vibration peak at 1645 cm^−1^ corresponding to the C=C group and the change in the stretching vibration peak at 3210 cm^−1^ corresponding to the O-H group. A Brookfield DV-S was used for the viscosity test.

### Calculation

Geometric optimization of the benzoyl radical reacting with SAE in the gas phase was computed using the B3LYP hybrid functional in conjunction with the 6-311 +  + G(d,p) basis set in Gaussian 09^27^. Frequency calculations were performed on the optimized geometries and the same basis set to ensure that the systems were local minima (no imaginary vibrational frequencies). The zero-point vibrational energies and thermal corrections to the Gibbs free energies at 298 K were extracted to obtain the thermodynamic property. The transition state was obtained using the QST2 method with one imaginary frequency. The reaction rate constant was provided by the Kinetic and Statistical Thermodynamical Package (KiSThelP)^28^. The reaction path degeneracy was set to 1.

### Cytotoxicity assay

HMPP was purified by distillation, and the result of element analysis was C 72.8% (73.1%) and H 7.44% (7.31%) for a colourless liquid. SAE mixed with 6% HMPP was cured for 30 minutes under UV irradiation. According to ISO 10993-5^29^, the curing product was immersed in serum containing medium at 0.1 g/ml after sterilization treatment for 24 h and 72 h at 37 °C. Then, 100 µl culture medium + 10% FBS containing 10^4^ of either of the two cell types was seeded into a well of a 96-well plate and cultivated under standard culture conditions for adhering cells to the plate. After a 24 h incubation, 10 µl cell culture fluid as extract was added to each well of the 96-well plate. Negative control wells were treated with 10 µl extract of high-density polyethylene (HDPE) (0.1 g/ml)^30^. A positive control was treated with 10 µl phenol solution (0.1 g/ml). A blank control consisted of serum containing medium. After 4 h, 24 h, 48 h and 72 h, 10 µl CCK-8 was added to the experimental wells, and after incubation for 4 hours, absorbance was measured using the Epoch (BioTek, USA).

### Adhesion ratio

The formulation (50 mg) was added to each well in a 96-well plate and cured for 1 minute under UV irradiation. Then, 100 µl 10% phosphate buffer solution as culture medium (PBS) containing 6 × 10^4^ cells (BRL3A or HEPG2) was seeded into a well of a 96-well plate and cultivated under standard culture conditions for adhering cells to the plate. After incubation for 24 h, the experimental group was rinsed three times with 100 μl PBS, while the control group was not rinsed with PBS, and the blank group was the solution without cells. Finally, all supernatants were removed and added to 100 μl culture medium and 10 μl CCK8. After incubation for 4 h, the absorbance was obtained with an Epoch instrument (BioTek, USA) at 450 nm. The adhesion rate is the fraction expressed as a quotient, in which the difference in the absorbance values of the experimental and blank groups is the numerator and the difference in the absorbance values of the control and blank group is the denominator.

### Hemostasis experiment *in vivo*

All mouse studies were approved by the review committee of Guangzhou Medical University. Three groups of animal experiments were performed in this study. Adult Sprague-Dawley (SD) rats (Guangdong Medical Lab Animal Center, China, n = 48) weighing 180–200 grams were used. The facility was maintained at 23 degrees Celsius, 35–45% relative humidity and a fixed l2-hour light 12-hour dark cycle. Procedures involving animals and their care were conducted in conformity with guidelines for the management and use of laboratory animals (Shanghai Science and Technology Press, published in 2012) and were approved by the Animal Care and Use Committee of Guangzhou Medical University. Forty-eight SD rats were randomly divided into four groups: blank control group, SAE + HMPP group, chitosan powder group and YBP group, with twelve rats in each group: six for the muscle hemostasis test and six for the liver hemostasis test. All rats received an intraperitoneal injection of 3% pentobarbital sodium at a dose of 45 mg/kg before the *in vivo* hemostasis experiment.

Muscle group: Rats were fixed in a prone position after anaesthesia was administered. Slicing through the rat skin, the left and right gluteus maxima were cut, and two bleeding wounds were formed with a scalpel (wound size 2 cm × 1 cm). The hemostatic material was immediately applied on the wound surface. Every 30 seconds, the condition of hemostasis was observed with filter paper until bleeding stopped. The standard of complete hemostasis is no red blood exudation within 3 minutes. The final hemostatic time was recorded and analysed.

Liver group: Rats were fixed in a supine position after anaesthesia was administered. Cutting a 2-cm longitudinal incision down the middle, with sterile gauze on the wound, the right hepatic lobe was gently squeezed out of the incision. With physiological saline gauze boosting and a fixed right hepatic lobe, an incision of approximately 1 cm × 1 cm was cut from the central part of the liver surface. Blood was immediately wiped off, and hemostatic material (SAE-HMPP/chitosan/YBP powder) was used to cover the incision. The standard of judging hemostasis was the same as for the muscle group. The complete hemostatic time was recorded, and data analysis was conducted.

All methods were performed in accordance with the relevant guidelines and regulations^31,32^.

## Electronic supplementary material

Supplementary material
