# Publisher Correction: Minimally Invasive Hemostatic Materials: Tackling a Dilemma of Fluidity and Adhesion by Photopolymerization *in situ*

**DOI:** 10.1038/s41598-020-70099-7

**Published:** 2020-08-14

**Authors:** Yun Zhang, Dandan Song, Hong Huang, Zhiling Liang, Houhe Liu, Yugang Huang, Cheng Zhong, Guodong Ye

**Affiliations:** 1 The Fifth Affiliated Hospital of Guangzhou Medical University, Guangzhou 510275, and Key Laboratory of Molecular Clinical Pharmacology, School of Pharmaceutical Sciences, Guangzhou Medical University, Guangzhou, 511436 People’s Republic of China; 2 College of Chemistry and Molecular Sciences, Wuhan University, Wuhan, 430072 People’s Republic of China

Correction to: *Scientific Reports* 10.1038/s41598-017-15368-8, published online 10 November 2017.

The original version of this Article contained extensive errors in the Reference list. References 6–30 were incorrectly listed as references 8–32 respectively and references 31–32 were incorrectly listed as references 6–7 respectively.

This has now been corrected in the PDF and HTML versions of the Article.
