# Supplementary material for: Minimally Invasive Hemostatic Materials: Tackling a Dilemma of Fluidity and Adhesion by Photopolymerization in situ
Source: Sci Rep. 2017 Nov 10;7:15250. doi: 10.1038/s41598-017-15368-8 (PMC5681561; doi:10.1038/s41598-017-15368-8)
Supplement: Supplementary file 1 — Supplementary material [file 41598_2017_15368_MOESM1_ESM.doc]

**Supplementary Information**

Title:

Minimally Invasive Haemostatic Materials: Tackling the Dilemma of Fluidity and Adhesion through Photopolymerization in situ

Author:

Yun ZHANG, Dandan SONG, Hong HUANG, Zhiling LIANG, Houhe LIU, Yugang HUANG, Cheng ZHONG, Guodong YE

**(Spectrum analysis of** **sucrose allyl ether)**

FT-IR (KBr pellet, cm^-1^): 1003 (ν C-O-C adjacent to the five-membered ring and six-membered rings), 1080 (ν C-O-C near to C=C group), 1644(ν C=C), 3210 (ν OH);

^1^H NMR (400MHz, CDCl_3_, δ ppm): 5.24 (d, 8H, CH_2_ on ethylene group), 5.89 (m, 8H, CH on ethylene group), 4.04 (d, 8H, aliphatic CH_2_ adjacent to C=C group), 3.38 (d, 8H, aliphatic CH_2_ adjacent to O-C-C=C group), 4.13 (m, 8H, five membered ring CH near to C=C group);

^13^C NMR (400MHz, CDCl_3_, δ ppm): 115.8 (CH_2_ on ethylene group), 131.7 (CH on ethylene group), 74.8 (aliphatic CH_2_ adjacent to C=C group), 70.2 (aliphatic CH_2_ adjacent to O-C-C=C group), 78.6 (five membered ring CH near to C=C group).

**(Gaussian 09 for quantitative calculation)**

basis Sets: uB3LYP/6-311++g(d,p)

Benzoyl Radical:

-------------------------------------------------------------------------------------------------------

Center Atomic Atomic Coordinates (Angstroms)

Number Number Type X Y Z

-------------------------------------------------------------------------------------------------------

1 6 0 1.277877 1.329961 0.000005

2 6 0 -0.090248 1.090395 0.000033

3 6 0 -0.563848 -0.231562 0.000033

4 6 0 0.336622 -1.301554 0.000004

5 6 0 1.706724 -1.055246 -0.000023

6 6 0 2.174943 0.258189 -0.000024

7 1 0 1.650213 2.348154 0.000014

8 1 0 -0.801425 1.908079 0.000044

9 1 0 -0.048635 -2.314648 0.000023

10 1 0 2.407270 -1.882054 -0.000052

11 1 0 3.242132 0.450048 -0.000046

12 6 0 -2.016965 -0.511098 0.000086

13 8 0 -2.925023 0.251989 -0.000084

-------------------------------------------------------------------------------------------------------

Sucrose Allyl Ether :

-------------------------------------------------------------------------------------------------------

Center Atomic Atomic Coordinates (Angstroms)

Number Number Type X Y Z

-------------------------------------------------------------------------------------------------------

1 6 0 3.765976 1.614093 -0.168711

2 6 0 3.525388 0.206254 0.416847

3 8 0 2.391641 -0.407725 -0.216113

4 6 0 1.164881 0.326004 -0.130597

5 6 0 2.481419 2.464288 -0.213621

6 6 0 1.306537 1.679236 -0.825472

7 6 0 3.429462 0.148430 1.941135

8 8 0 4.333859 1.520872 -1.467339

9 8 0 2.181858 2.859521 1.126690

10 8 0 1.570068 1.516464 -2.217363

11 6 0 -0.527604 -1.917814 1.036292

12 6 0 -1.915978 -1.267539 1.120916

13 6 0 -2.487732 -1.599991 -0.281826

14 8 0 -1.371393 -2.002215 -1.109969

15 6 0 -0.153051 -1.742084 -0.462457

16 8 0 -1.789394 0.120675 1.382466

17 8 0 0.339669 -1.435129 2.028526

18 6 0 0.902473 -2.768254 -0.937828

19 6 0 -3.215659 -0.445990 -0.962151

20 8 0 0.203054 -0.394445 -0.845215

21 8 0 -4.243645 -0.000962 -0.077964

22 8 0 1.581354 -2.411075 -2.119090

23 8 0 3.218757 -1.226305 2.308441

24 1 0 4.496757 2.140097 0.451015

25 1 0 4.367444 -0.417508 0.110341

26 1 0 0.867858 0.459912 0.909212

27 1 0 2.685535 3.337755 -0.842165

28 1 0 0.372811 2.239572 -0.678049

29 1 0 2.623385 0.776692 2.325152

30 1 0 4.377383 0.512824 2.354062

31 1 0 3.616852 1.324512 -2.089050

32 1 0 1.429804 3.460985 1.121084

33 1 0 1.005399 0.793810 -2.528592

34 1 0 -0.679021 -3.003049 1.151184

35 1 0 -2.517988 -1.739148 1.903953

36 1 0 -3.158412 -2.461525 -0.215756

37 1 0 -2.684481 0.483783 1.349065

38 1 0 1.257875 -1.714342 1.880588

39 1 0 1.615045 -2.940541 -0.121995

40 1 0 0.372937 -3.701379 -1.137306

41 1 0 -2.507445 0.362856 -1.172074

42 1 0 -3.644976 -0.791430 -1.910827

43 1 0 2.162126 -1.675860 -1.881285

44 1 0 3.267501 -1.293449 3.267511

45 6 0 -5.109136 0.975317 -0.660180

46 1 0 -5.566766 0.548629 -1.565985

47 1 0 -4.536385 1.863548 -0.961245

48 6 0 -6.168324 1.338971 0.332901

49 1 0 -6.734833 0.504600 0.739035

50 6 0 -6.445696 2.584872 0.706589

51 1 0 -5.889170 3.433245 0.319525

52 1 0 -7.243587 2.803248 1.407017

-------------------------------------------------------------------------------------------------------

Product:

-------------------------------------------------------------------------------------------------------

Center Atomic Atomic Coordinates (Angstroms)

Number Number Type X Y Z

-------------------------------------------------------------------------------------------------------

1 6 0 4.983052 2.740438 -0.268645

2 6 0 5.331908 1.347830 0.297448

3 8 0 4.490071 0.344484 -0.293288

4 6 0 3.081937 0.550440 -0.136535

5 6 0 3.470106 3.031738 -0.236696

6 6 0 2.658518 1.855893 -0.809692

7 6 0 5.347950 1.254516 1.822396

8 8 0 5.476423 2.874925 -1.593813

9 8 0 3.110176 3.280174 1.123829

10 8 0 2.895421 1.807442 -2.214790

11 6 0 2.480511 -2.197339 1.031520

12 6 0 0.959286 -2.140431 1.227453

13 6 0 0.458511 -2.638213 -0.153615

14 8 0 1.583280 -2.565773 -1.062303

15 6 0 2.649785 -1.865898 -0.477362

16 8 0 0.557791 -0.818227 1.549296

17 8 0 3.161425 -1.429284 1.988874

18 6 0 3.984577 -2.393435 -1.056347

19 6 0 -0.704189 -1.839962 -0.734716

20 8 0 2.432339 -0.479432 -0.823215

21 8 0 -1.746607 -1.827216 0.239480

22 8 0 4.399560 -1.768520 -2.248531

23 8 0 5.729930 -0.088413 2.165715

24 1 0 5.486694 3.505680 0.327569

25 1 0 6.331932 1.097172 -0.062081

26 1 0 2.809125 0.557410 0.918521

27 1 0 3.291099 3.917300 -0.855870

28 1 0 1.589419 2.013442 -0.609625

29 1 0 4.378375 1.502660 2.258977

30 1 0 6.088608 1.970427 2.197992

31 1 0 4.860624 2.415938 -2.184836

32 1 0 2.182139 3.533691 1.166121

33 1 0 2.634216 0.924086 -2.514153

34 1 0 2.771432 -3.257154 1.109109

35 1 0 0.644581 -2.826045 2.020643

36 1 0 0.174975 -3.692498 -0.087973

37 1 0 -0.409235 -0.829364 1.553435

38 1 0 4.101844 -1.324017 1.772782

39 1 0 4.758971 -2.301635 -0.284786

40 1 0 3.840581 -3.452189 -1.277864

41 1 0 -0.373308 -0.820990 -0.963627

42 1 0 -1.047632 -2.312359 -1.663026

43 1 0 4.670228 -0.874153 -2.001060

44 1 0 5.817799 -0.142553 3.122779

45 6 0 -2.962930 -1.239631 -0.234493

46 1 0 -3.277370 -1.754209 -1.158079

47 1 0 -2.794407 -0.184282 -0.503205

48 6 0 -4.006772 -1.362986 0.811835

49 1 0 -4.049392 -2.295991 1.362276

50 6 0 -5.107418 -0.372680 0.958792

51 1 0 -4.703692 0.650520 1.018051

52 1 0 -5.648425 -0.540371 1.894363

53 6 0 -9.482878 1.174490 -1.000412

54 6 0 -8.313963 0.428722 -1.085465

55 6 0 -7.366626 0.468873 -0.051854

56 6 0 -7.615480 1.275288 1.067224

57 6 0 -8.785702 2.026331 1.149383

58 6 0 -9.721195 1.975588 0.117796

59 1 0 -10.209896 1.133951 -1.803535

60 1 0 -8.111118 -0.196291 -1.946317

61 1 0 -6.900651 1.328148 1.878810

62 1 0 -8.966923 2.649028 2.017939

63 1 0 -10.633070 2.558432 0.184110

64 6 0 -6.124305 -0.358930 -0.202263

65 8 0 -5.914260 -0.989864 -1.218998

-------------------------------------------------------------------------------------------------------

TS:

-------------------------------------------------------------------------------------------------------

Center Atomic Atomic Coordinates (Angstroms)

Number Number Type X Y Z

-------------------------------------------------------------------------------------------------------

1 6 0 -9.935680 1.010729 -0.643556

2 6 0 -8.798725 0.261408 -0.917454

3 6 0 -7.624960 0.472762 -0.175618

4 6 0 -7.604425 1.440387 0.834602

5 6 0 -8.743603 2.196389 1.097792

6 6 0 -9.908088 1.979392 0.362682

7 1 0 -10.843405 0.845467 -1.212831

8 1 0 -8.799058 -0.489873 -1.698249

9 1 0 -6.699954 1.604989 1.405809

10 1 0 -8.724850 2.950295 1.876121

11 1 0 -10.796315 2.564543 0.572976

12 6 0 -6.422732 -0.344402 -0.477051

13 8 0 -6.266290 -1.079002 -1.396824

14 6 0 4.968849 2.777724 -0.369922

15 6 0 5.343534 1.425044 0.271542

16 8 0 4.540475 0.371219 -0.283597

17 6 0 3.124349 0.545864 -0.161917

18 6 0 3.448407 3.029205 -0.378444

19 6 0 2.678515 1.804596 -0.905152

20 6 0 5.331520 1.408952 1.799265

21 8 0 5.483033 2.860134 -1.691524

22 8 0 3.057830 3.335850 0.961463

23 8 0 2.940911 1.694119 -2.302393

24 6 0 2.558994 -2.141538 1.144774

25 6 0 1.032750 -2.102161 1.303469

26 6 0 0.574026 -2.691119 -0.056834

27 8 0 1.719298 -2.650833 -0.942389

28 6 0 2.756385 -1.893310 -0.377126

29 8 0 0.599659 -0.771826 1.535687

30 8 0 3.205142 -1.308820 2.072100

31 6 0 4.115172 -2.422372 -0.895299

32 6 0 -0.586775 -1.949036 -0.711730

33 8 0 2.517169 -0.534618 -0.808170

34 8 0 -1.651876 -1.890274 0.235029

35 8 0 4.543552 -1.858366 -2.112966

36 8 0 5.742061 0.096145 2.218390

37 1 0 5.440965 3.585036 0.195892

38 1 0 6.356930 1.184737 -0.056316

39 1 0 2.831225 0.595494 0.886342

40 1 0 3.257361 3.877339 -1.044737

41 1 0 1.602290 1.942422 -0.730300

42 1 0 4.347074 1.652057 2.204028

43 1 0 6.045070 2.163041 2.151924

44 1 0 4.889547 2.357795 -2.269944

45 1 0 2.119321 3.551131 0.977965

46 1 0 2.708485 0.790312 -2.561827

47 1 0 2.866298 -3.189664 1.288225

48 1 0 0.711941 -2.745716 2.128829

49 1 0 0.307770 -3.744857 0.064807

50 1 0 -0.367113 -0.801390 1.518364

51 1 0 4.147628 -1.196555 1.868946

52 1 0 4.869678 -2.267552 -0.114326

53 1 0 3.999825 -3.495301 -1.057303

54 1 0 -0.264110 -0.941517 -0.996531

55 1 0 -0.901556 -2.483585 -1.616544

56 1 0 4.784862 -0.943818 -1.913458

57 1 0 5.813125 0.094036 3.178377

58 6 0 -2.863094 -1.344983 -0.305301

59 1 0 -3.162414 -1.949729 -1.176160

60 1 0 -2.688822 -0.319129 -0.660359

61 6 0 -3.925451 -1.371041 0.739629

62 1 0 -4.116967 -2.335414 1.201069

63 6 0 -4.737746 -0.308278 1.015129

64 1 0 -4.452303 0.688633 0.691995

65 1 0 -5.417574 -0.358326 1.857877

-------------------------------------------------------------------------------------------------------
